# Impact of the size of the normal database on the performance of the specific binding ratio in dopamine transporter SPECT

**DOI:** 10.1186/s40658-020-00304-z

**Published:** 2020-05-20

**Authors:** Helen Schmitz-Steinkrüger, Catharina Lange, Ivayla Apostolova, Holger Amthauer, Wencke Lehnert, Susanne Klutmann, Ralph Buchert

**Affiliations:** 1 Department for Diagnostic and Interventional Radiology and Nuclear Medicine, University Hospital Hamburg-Eppendorf, Hamburg, Germany; 2 Department of Nuclear Medicine, Charité - Universitätsmedizin Berlin, Corporate Member of Freie Universität Berlin, Humboldt-Universität zu Berlin, and Berlin Institute of Health, Berlin, Germany

**Keywords:** Dopamine transporter, SPECT, FP-CIT, Specific binding ratio, Normal database

## Abstract

**Background:**

This study investigated the impact of the size of the normal database on the classification performance of the specific binding ratio (SBR) in dopamine transporter (DAT) SPECT with [^123^I]FP-CIT in different settings.

**Methods:**

The first subject sample comprised 645 subjects from the Parkinson’s Progression Marker Initiative (PPMI), 207 healthy controls (HC), and 438 Parkinson’s disease (PD) patients. The second sample comprised 372 patients from clinical routine patient care, 186 with non-neurodegenerative parkinsonian syndrome (PS) and 186 with neurodegenerative PS. Single-photon emission computed tomography (SPECT) images of the clinical sample were reconstructed with two different reconstruction algorithms (filtered backprojection, iterative ordered subsets expectation maximization (OSEM) reconstruction with resolution recovery). The putaminal specific binding ratio (SBR) was computed using an anatomical region of interest (ROI) predefined in standard (MNI) space in the Automated Anatomic Labeling (AAL) atlas or using hottest voxels (HV) analysis in large predefined ROIs. SBR values were transformed to z-scores using mean and standard deviation of the SBR in a normal database of varying sizes (*n* = 5, 10, 15,…, 50) randomly selected from the HC subjects (PPMI sample) or the patients with non-neurodegenerative PS (clinical sample). Accuracy, sensitivity, and specificity for identifying patients with PD or neurodegenerative PS were determined as performance measures using a predefined fixed cutoff on the z-score. This was repeated for 10,000 randomly selected normal databases, separately for each size of the normal database. Mean and 5th percentile of the performance measures over the 10,000 realizations were computed. Accuracy, sensitivity, and specificity when using the whole set of HC or non-neurodegenerative PS subjects as normal database were used as benchmark.

**Results:**

Mean loss of accuracy of the putamen SBR z-score was below 1% when the normal database included at least 15 subjects, independent of subject sample (PPMI or clinical), reconstruction method (filtered backprojection or OSEM), and ROI method (AAL or HV). However, the variability of the accuracy of the putamen SBR z-score decreased monotonically with increasing size of normal database and was still considerable at size 15. In order to achieve less than 5% “maximum” loss of accuracy (defined by the 5th percentile) in all settings required at least 25 to 30 subjects in the normal database. Reduction of mean and “maximum” loss of accuracy of the putamen SBR z-score by further increasing the size of the normal database was very small beyond size 40.

**Conclusions:**

The results of this study suggest that 25 to 30 is the minimum size of the normal database to reliably achieve good performance of semi-quantitative analysis in dopamine transporter (DAT) SPECT, independent of the algorithm used for image reconstruction and the ROI method used to estimate the putaminal SBR.

## Introduction

Single-photon emission computed tomography (SPECT) with N-ω-fluoropropyl-2β-carbomethoxy-3β-(4-I-123-iodophenyl)nortropane (FP-CIT) is widely used for the detection (or exclusion) of nigrostriatal degeneration in clinically uncertain parkinsonian syndromes (PS) [1–4]. Visual reading of the FP-CIT SPECT images can be complemented by semi-quantitative analysis using the specific binding ratio (SBR) to characterize FP-CIT binding to the dopamine transporter (DAT) in the striatum and striatal subregions [5–10].

SBR analysis is sensitive to site- and/or camera-specific variability of SPECT image characteristics caused by differences in acquisition and reconstruction protocols, which limits sharing of normal databases and SBR cutoff values between sites and/or cameras [5, 11–18]. In prospective studies, this problem can be addressed by harmonization of acquisition protocols and centralized image reconstruction in an imaging core lab [15–17, 19]. This is difficult to realize in everyday clinical patient care so that the use of a camera-specific normal database often is the most straightforward solution in clinical routine. This is facilitated by the fact that generation of a camera-specific normal database does not necessarily require prospective scanning of healthy subjects. FP-CIT SPECT images from patients with clinically uncertain PS that have been interpreted normal in clinical routine might be used retrospectively for the normal database.

This raises the question about the impact of the size of the normal database on the performance of semi-quantitative analysis. Of particular interest is the minimum size of the normal database required for good performance of SBR analysis.

More complex methods including convolutional neural networks have been proposed for automatic classification of FP-CIT SPECT [18, 20, 21]. However, conventional SBR analysis is still widely used because it is easy to understand (no black box) and achieves high accuracy provided that an appropriate normal database is used. Furthermore, more complex methods such as convolutional neural networks usually require considerably larger databases for training and validation than univariate SBR analysis. Thus, SBR analysis most likely will continue to play a role in FP-CIT SPECT in the future.

The aim of the present study, therefore, was to analyze the impact of the size of the normal database on the performance of SBR analysis of FP-CIT SPECT in different settings, that is, for two different patient samples, two different reconstruction algorithms, and two different region-of-interest (ROI) methods to estimate the SBR.

## Materials and methods

### Parkinson’s Progression Markers Initiative (PPMI) sample

The first sample of FP-CIT SPECT images used in this study was obtained from the PPMI (http://www.ppmi-info.org/data) [19]. It comprised the baseline FP-CIT scans of 645 FP-subjects, 207 healthy control (HC) subjects and 438 Parkinson’s disease (PD) patients. Up-to-date information on the PPMI is given at http://www.ppmi-info.org. The PPMI is a longitudinal, multi-center study that aims to assess the progression of clinical features, imaging, and biologic markers in patients with PD and HC subjects. Details of the PPMI eligibility criteria are given at http://www.ppmi-info.org/wp-content/uploads/2014/01/PPMI-AM7-Protocol.pdf. Details of the PPMI FP-CIT SPECT protocol are given at http://www.ppmi-info.org/study-design/research-documents-and-sops/ [19]. Raw projection data had been transferred to the PPMI imaging core lab for central image reconstruction using an iterative ordered subsets expectation maximization (OSEM) algorithm with eight iterations and eight subsets and no filtering on a Hermes workstation [22]. Post-reconstruction attenuation correction according to Chang [23] had been performed using a site-specific attenuation coefficient derived from phantom measurements performed during site initiation for the PPMI [24]. A three-dimensional Gaussian filter with 6-mm full width at half maximum had been applied after attenuation correction [24]. No scatter correction had been performed [22].

### Clinical sample

Three-hundred-and-seventy-two patients from routine clinical patient care were recruited retrospectively from the database of the University Medical Center Hamburg-Eppendorf. The patients were categorized into “neurodegenerative PS” and “non-neurodegenerative PS”. The neurodegenerative group (*n* = 186, 45.7% females, 65.9 ± 10.4 years) comprised the Lewy body disease spectrum including PD, PD dementia and dementia with Lewy bodies, and atypical Parkinsonian syndromes including multiple systems atrophy, progressive supranuclear palsy, and corticobasal degeneration. The non-neurodegenerative group (*n* = 186, 52.2% females, 65.5 ± 12.5 years) comprised essential tremor, drug-induced parkinsonism, several types of dystonia, psychogenic parkinsonism, and various other diagnoses not associated with nigrostriatal degeneration. The clinical diagnoses as standard of truth were taken from the written report of a movement disorder specialist in the patient’s file at least 12 months after FP-CIT SPECT in all 186 patients with neurodegenerative PS (mean follow-up 41 ± 22 months, range 12–95 months) and in 44 of the patients with non-neurodegenerative PS (mean follow-up 38 ± 22 months, 13–97 months). The remaining patients with non-neurodegenerative PS had less than 12 months follow-up and were included to increase sample size and to avoid imbalance with respect to group size (neurodegenerative versus non-neurodegenerative).

FP-CIT SPECT had been performed according to common guidelines [25] with a double-head SPECT system (Siemens Symbia T2 or Siemens E.CAM). In order to ensure consistent image reconstruction in all patients, projection data were retrieved from the archive and reconstructed retrospectively. Two different reconstruction algorithms were used in all patients. First, SPECT images were reconstructed using filtered backprojection implemented in the SPECT system software (Butterworth filter of the 5th order with cutoff 0.6 cycles/pixel). Uniform post reconstruction attenuation correction was performed according to Chang’s method (*μ* = 0.12/cm) [23]; scatter correction was not performed. Second, SPECT images were reconstructed using the OSEM algorithm with resolution recovery implemented in the HybridRecon-Neurology tool of the Hermes SMART workstation v1.6 with parameter settings recommended for FP-CIT SPECT by Hermes (effective number of iterations 80, postfiltering with three-dimensional Gaussian kernel of 7-mm full width at half maximum, uniform attenuation correction with narrow-beam attenuation coefficient 0.146/cm, simulation-based scatter correction, resolution recovery with a Gaussian model).

**Fig. 1:**
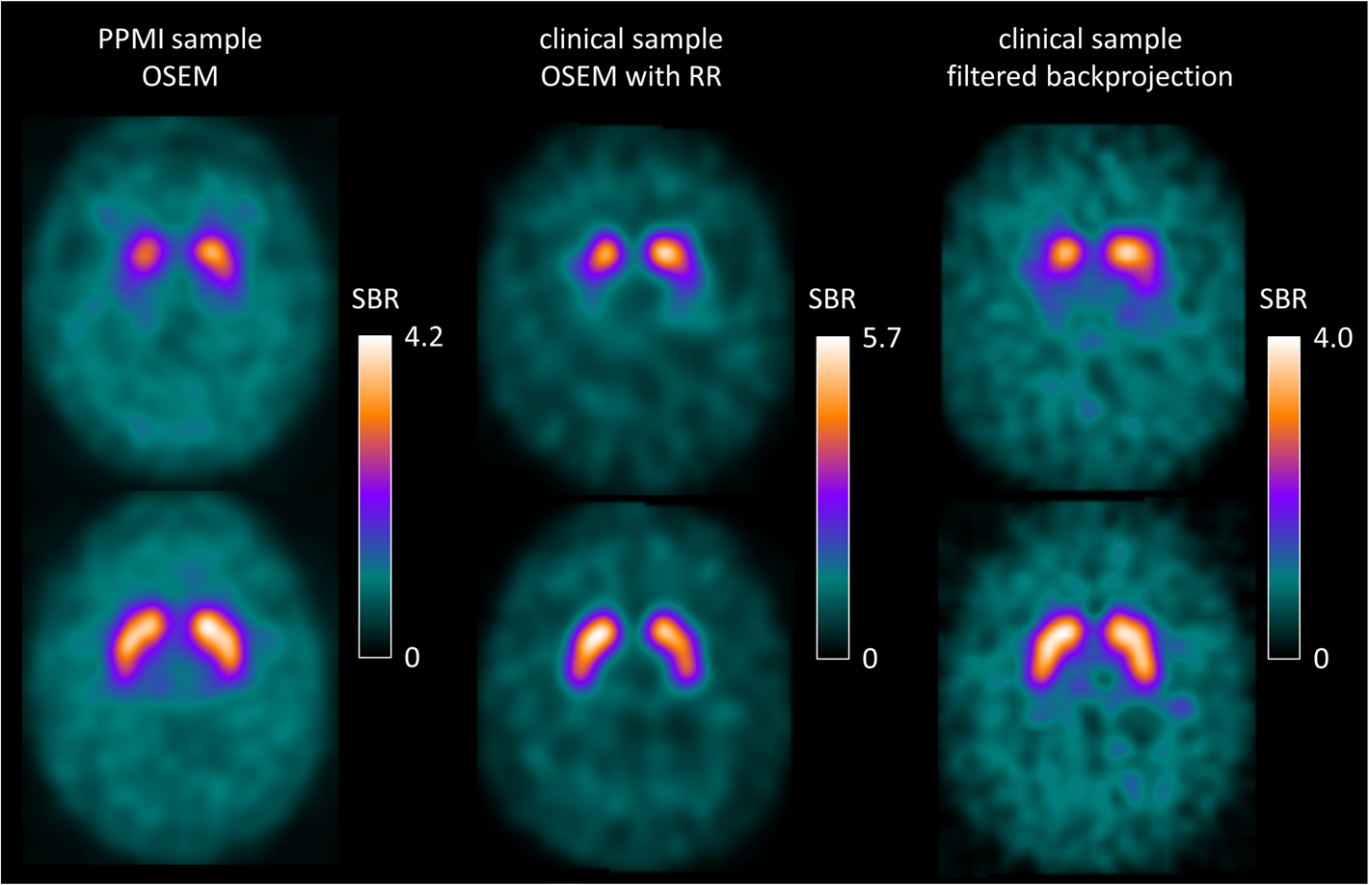
Representative FP-CIT SPECT images. The left column shows a patient with Parkinson’s disease (top) and a healthy control subject (bottom) from the PPMI sample. The middle and right columns show the same patient with neurodegenerative PS (top) and the same patient with non-neurodegenerative PS (bottom) from the clinical sample reconstructed with iterative reconstruction (ordered subsets expectation maximization, OSEM) with resolution recovery (RR; middle column) or with filtered backprojection (right column). Shown are 4-mm-thick transaxial slices in MNI space with voxel intensities scaled to the individual 75th percentile of the voxel intensity in the reference region. The upper threshold of the colour table was adjusted separately for each of the three settings

Representative FP-CIT SPECT images from the different settings are shown in Fig. 1.

### Semi-quantitative SBR analysis

Individual SPECT images were normalized (affine) to a custom-made FP-CIT template in the anatomical space of the Montreal Neurological Institute (MNI) using the Statistical Parametric Mapping software package (version SPM12) [26]. Voxel intensities were scaled voxel-wise to the 75th percentile of the voxel intensity in a reference region comprising the whole brain except the striata, thalamus, brain stem, and ventricles [27, 28].

The conventional unilateral putamen SBR was computed by applying anatomical ROIs predefined in MNI space by the Automatic Anatomical Labeling atlas (AAL) [29]. The mean value of the scaled voxel intensity in the AAL ROI was used to calculate the conventional SBR (= mean scaled voxel intensity in the ROI − 1).

In addition, hottest voxels (HV) analysis was performed using large unilateral ROIs predefined in MNI space [30]. The ROIs for HV analysis were much bigger than the actual putamen volume in order to guarantee that all counts originating from the putamen were included. The number of hottest voxels to be averaged for the unilateral putamen was fixed to a volume of 10 ml. The hottest voxel SBR (HV-SBR) was calculated as mean scaled voxel intensity in the 10-ml hottest ROI voxels − 1.

SBR analysis was restricted to the putamen, and the minimum of the unilateral putamen SBR of left and right hemispheres was used in all further analyses. The rationale for this was that the effect size of the reduction in PD in general is larger in the bilateral putamen compared to the bilateral caudate, and larger in the contralateral putamen compared to the ipsilateral putamen [31]. Other conventional semi-quantitative parameters such as putamen-to-caudate ratio and left-right asymmetry were not considered because they did not provide additional information beyond the putamen SBR (Appendix).

### Statistical analysis

Normal databases of sizes *n* = 5, 10, 15,…, 50 were obtained by randomly selecting the appropriate number of HC subjects (in case of the PPMI sample) or patients with non-neurodegenerative PS (in case of the clinical sample). Mean and standard deviation of the SBR in the resulting normal database was used to transform individual SBR values to z-scores using the following formula: z-score = (individual SBR − mean SBR in normal database) / standard deviation of SBR in normal database. Overall accuracy, sensitivity, and specificity of the z-score to identify patients with PD (PPMI sample) or neurodegenerative PS (clinical sample) were computed using a z-score of −2.5 as cutoff. The whole sample was used as test set in all cases, that is, the test set comprised all 645 PPMI subjects or all 372 clinical patients, independent of the (size of) the normal database. This was repeated for 10,000 randomly selected normal databases for each size of the normal database. The mean and the 5th percentile of overall accuracy, specificity, and sensitivity were computed over the 10,000 repeats. The performance of the z-score obtained with all HC subjects or all patients with non-neurodegenerative PS as normal database was used as benchmark.

## Results

**Fig. 2:**
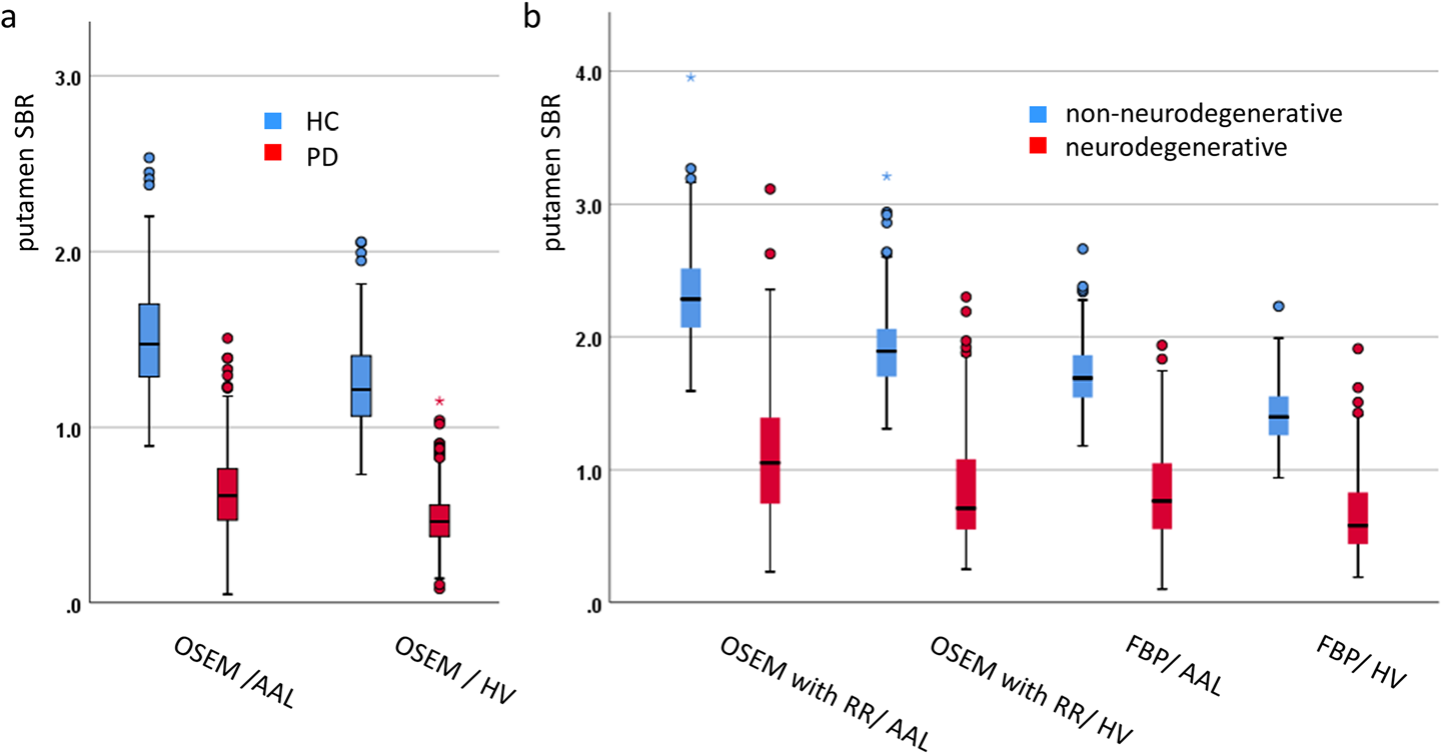
Box-and-whisker-plot of putamen SBR in healthy control (HC) subjects versus patients with Parkinson’s disease (PD) of the PPMI (**a**) and patients with non-neurodegenerative PS versus patients with neurodegenerative PS of the clinical sample (**b**) for different ROI methods to estimate the putamen SBR (conventional analysis with AAL ROI versus hottest voxels (HV) analysis). Different algorithms to reconstruct the SPECT images (iterative ordered subsets expectation maximization (OSEM) reconstruction with resolution recovery (RR) versus filtered backprojection (FBP)) were tested in the clinical sample only

Box plots of the putamen SBR for the different settings are shown in Fig. 2. The absolute value of the SBR strongly depended on the reconstruction algorithm and on the method used to estimate the SBR.

**Fig. 3:**
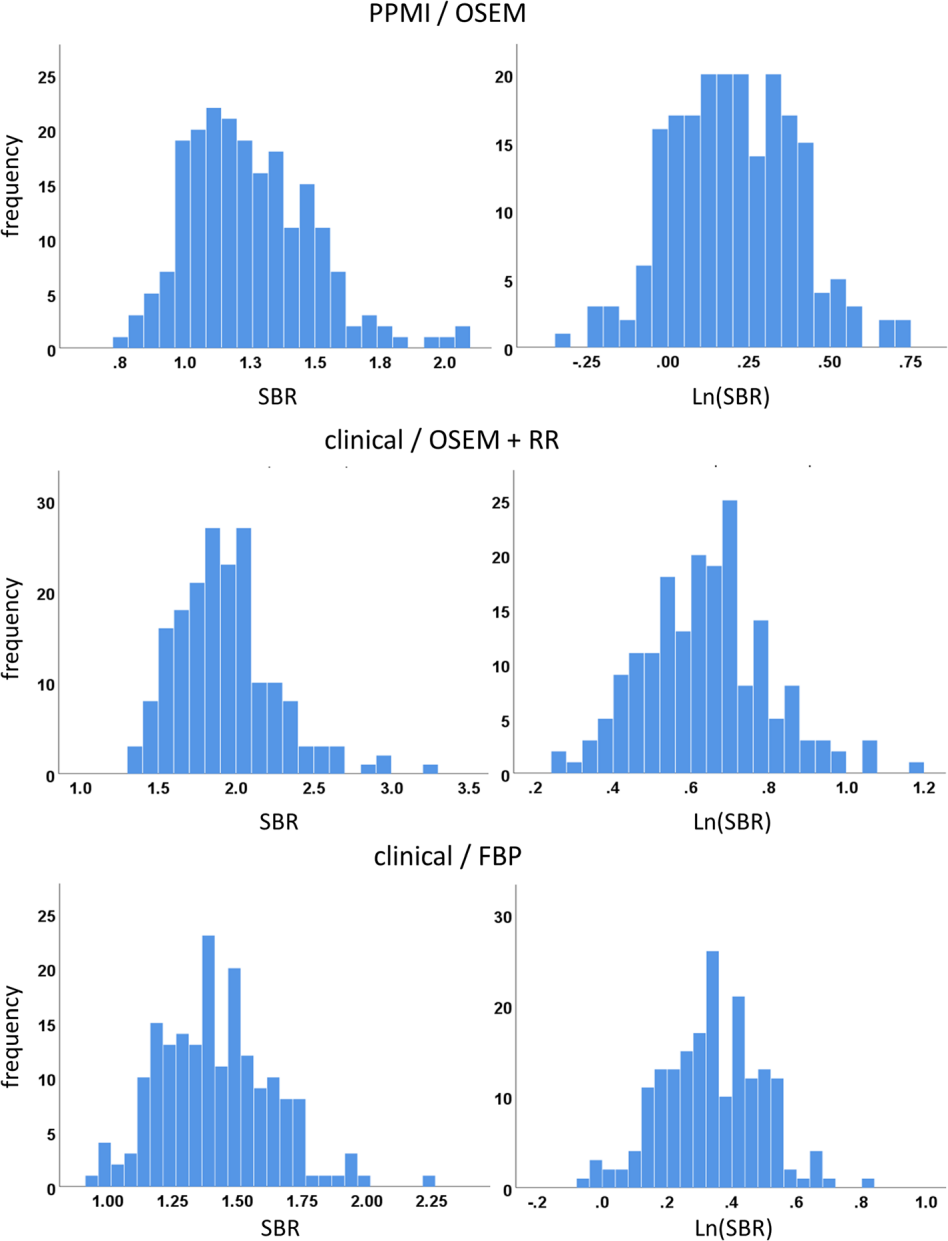
Histogram of the putamen hottest voxels SBR before (left column) and after (right column) Ln-transformation in the healthy controls of the PPMI sample with ordered subsets expectation maximization (OSEM) reconstruction (top row) and in the patients with non-neurodegenerative PS of the clinical sample with OSEM reconstruction and resolution recovery (RR, middle row) or with filtered backprojection (bottom row)

Histograms of putamen SBR in HC subjects or patients with non-neurodegenerative PS showed slightly skewed distributions, mainly due to extended tails towards high SBR values (Fig. 3). The skewness of the SBR distribution was significantly different from zero in all settings. The skewness was largest in the patients with non-neurodegenerative PS of the clinical sample with OSEM reconstruction with resolution recovery (skewness = 0.877 and 0.908 for AAL-SBR and HV-SBR, respectively). The skewness was smallest in the patients with non-neurodegenerative PS of the clinical sample with filtered backprojection (skewness = 0.560 and 0.455 for AAL-SBR and HV-SBR, respectively). In order to account for the skewness of the SBR distributions, SBR values were Ln-transformed prior to transforming them to z-scores (Fig. 3). This was done in all settings.

**Fig. 4:**
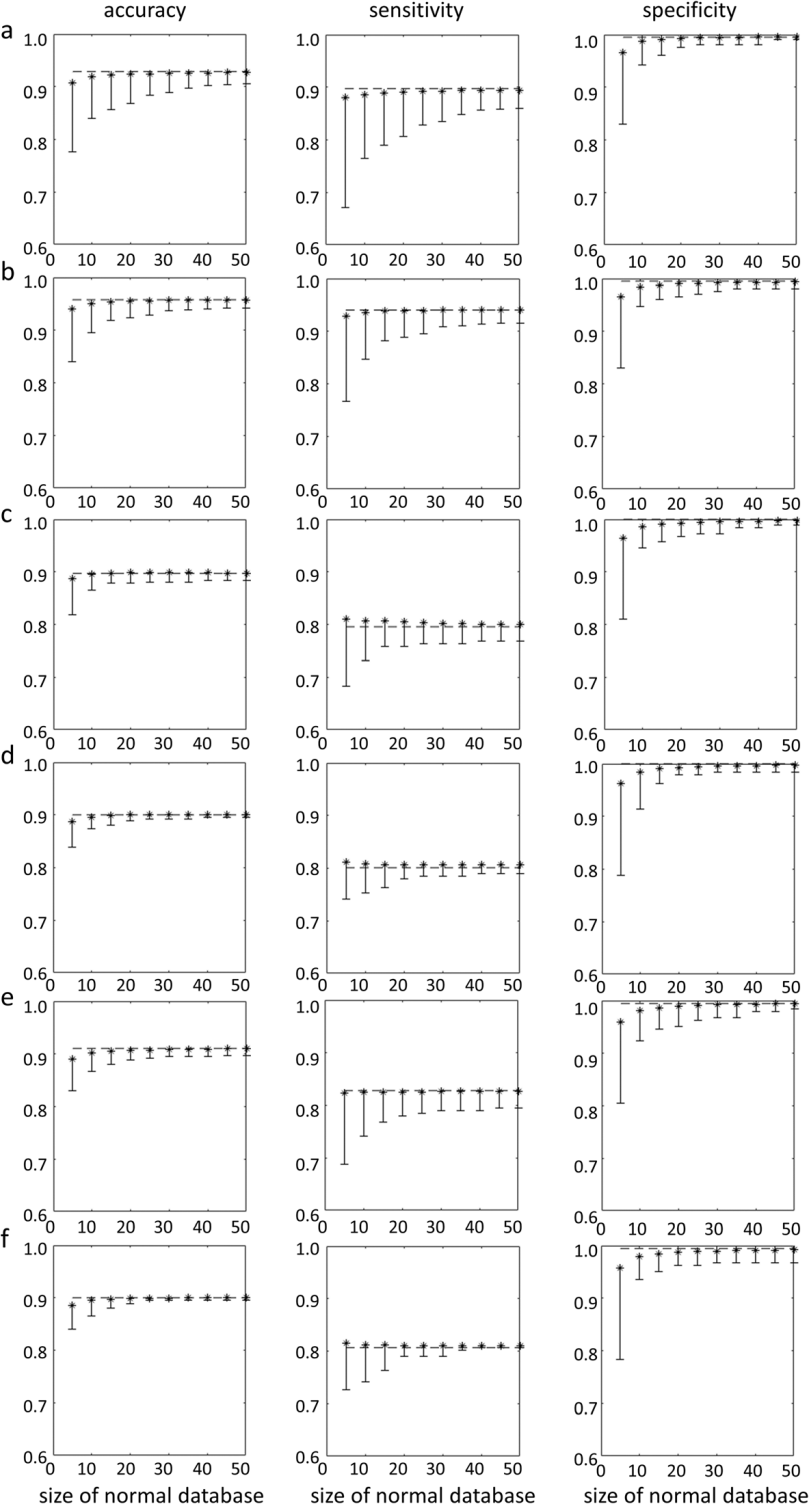
Mean accuracy (left column), sensitivity (middle column), and specificity (right column) of the putamen SBR z-score for identification of PD patients or patients with neurodegenerative PS as a function of the size of the normal database used to estimate mean and standard deviation of normal putamen SBR for transforming SBR values into z-scores (**a** PPMI sample, OSEM, AAL-SBR; **b** PPMI sample, OSEM, HV-SBR; **c** clinical sample, OSEM with resolution recovery, AAL-SBR; **d** clinical sample, OSEM with resolution recovery, HV-SBR; **e** clinical sample, filtered backprojection, AAL-SBR; **f** clinical sample, filtered backprojection, HV-SBR). SBR values were Ln-transformed prior to transforming them into z-scores in all settings. The error bars indicate the difference between mean accuracy, sensitivity, or specificity and the 5th percentile over the 10,000 randomly sampled normal databases. The dashed line represents the performance of the z-score of the putamen SBR when all HC subjects (*n* = 207) or all patients with non-neurodegenerative PS (*n* = 186) were used to estimate mean and standard deviation of normal putamen SBR for transforming SBR values into z-scores as benchmark

Figure 4 shows the impact of the size of the normal database on overall accuracy, sensitivity, and specificity of the putamen SBR z-score to identify patients with PD in the PPMI sample or patients with neurodegenerative PS in the clinical sample.

**Fig. 5:**
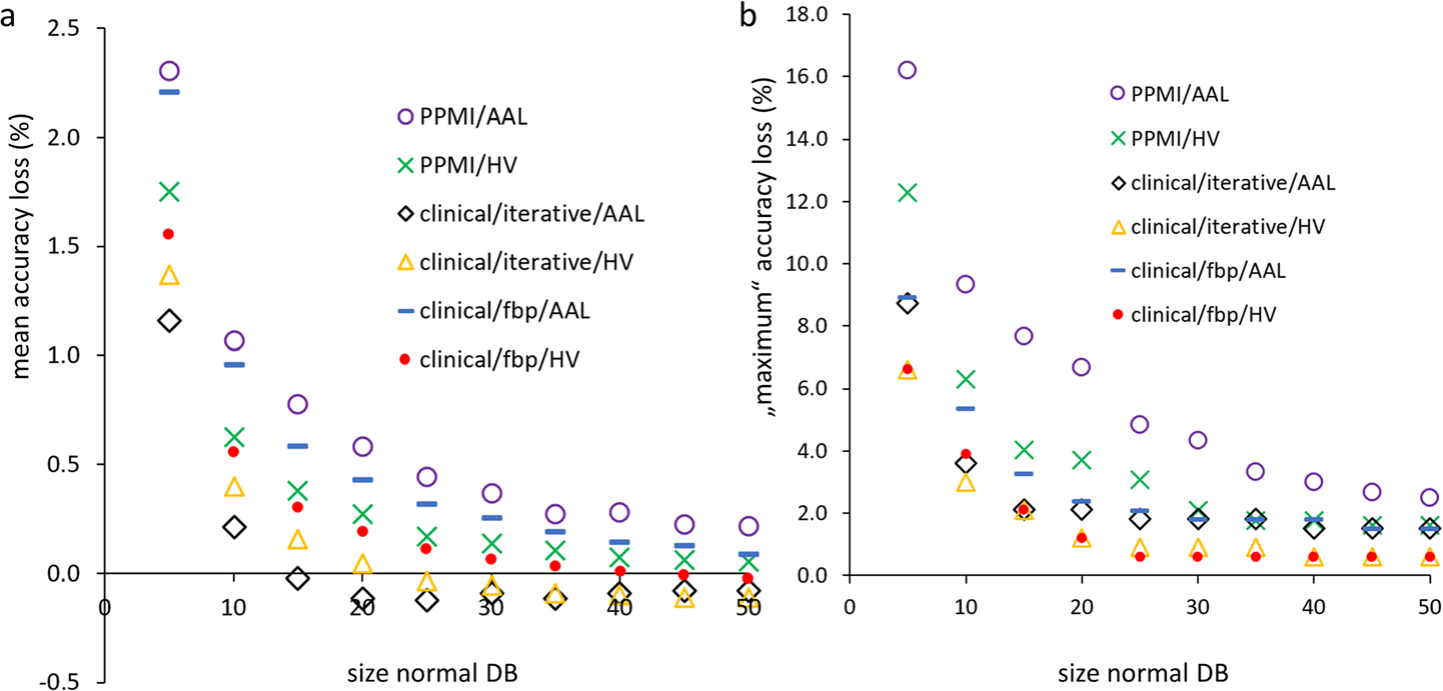
Mean relative loss of accuracy (**a**) and “maximum” relative loss of accuracy (**b**) of the putamen SBR z-score for differentiation between PD patients and HC subjects of the PPMI or between patients with neurodegenerative PS and patients with non-neurodegenerative PS of the clinical sample as a function of size of the normal database (DB) used to estimate mean and standard deviation of normal putamen SBR for transforming SBR values into z-scores. SBR values were Ln-transformed prior to transforming them into z-scores in all settings. The relative loss of accuracy was computed with respect to the benchmark accuracy (normal database comprising all HC subjects or all patients with non-neurodegenerative PS), that is, relative accuracy loss (%) = 100 * (benchmark accuracy − accuracy) / benchmark accuracy. The maximum accuracy loss corresponds to the 5th percentile of the accuracy estimates over the 10,000 random samples to generate the normal database.

Mean relative loss of accuracy and “maximum” relative loss of accuracy of the putamen SBR z-score for differentiation between PD patients and HC subjects of the PPMI or between patients with neurodegenerative PS and patients with non-neurodegenerative PS of the clinical sample as a function of size of the normal database are given in Fig. 5.

## Discussion

Mean loss of overall accuracy of the z-score of the (Ln-transformed) putamen SBR was below 1% when the normal database included at least 15 subjects, independent of the subject sample, the reconstruction method, and the ROI method (Fig. 5a). However, the variability of accuracy, sensitivity, and specificity of the putamen SBR z-score decreased monotonically with increasing size of the normal database and was still considerable at size 15 (Fig. 4). The “maximum” loss of accuracy of the putamen SBR z-score was less than 5% when the normal database included at least 25 to 30 subjects (Fig. 5b). This suggests that a normal database for SBR analysis in DAT SPECT in clinical routine should include at least 25 to 30 subjects.

Reduction of mean and “maximum” loss of accuracy of the putamen SBR z-score by further increasing the size of the normal database was very small beyond size 40 (Fig. 5). This suggests that a normal database including 40 subjects provides close-to-optimal performance of putaminal SBR in DAT SPECT.

Sensitivity was lower than specificity in all settings (Fig. 4). This was due to the rather conservative cutoff of −2.5 on the z-score for classification of FP-CIT SPECT images. Sensitivity can be increased by using a less conservative cutoff which, however, will result in the reduction of specificity. In the absence of disease-modifying treatment for neurodegenerative PS, the trade-off between sensitivity and specificity is usually balanced in favor of high specificity in clinical routine. The use of a rather conservative cutoff in this study is in line with this.

Lower overall accuracy of the putamen SBR in the clinical sample relative to the PPMI sample was mainly driven by reduced sensitivity in the clinical sample (Fig. 4). Visual inspection of the false-negative clinical SPECT images confirmed the SBR-based classification in most cases. Thus, most of the false-negative cases in the clinical sample were subjects without evidence of dopaminergic deficit (SWEDD). Several studies suggest that the majority of SWEDD patients do not have a neurodegenerative PS [32, 33]. Reduced sensitivity of the putamen SBR in the clinical sample therefore most likely was due to clinical overdiagnosis of neurodegenerative PS at clinical follow-up used as standard of truth in this study [34]. The PPMI sample did not include SWEDD subjects, as the PPMI handles SWEDD as a separate category, different from healthy controls and PD patients (s. PPMI study protocol at http://www.ppmi-info.org/wp-content/uploads/2018/02/PPMI-AM-13-Protocol.pdf). The lack of SWEDD patients in the PPMI sample explains the lower sensitivity of the putamen SBR z-score in the clinical sample at least to some extent. Thus, the findings in the clinical sample support the use of a normal database comprised of patients with a non-neurodegenerative parkinsonian syndrome. This is practically relevant because prospective scanning of healthy subjects constitutes a major obstacle at many sites, particularly in smaller hospitals and private practices.

The findings with respect to the impact of the size of the normal database on classification performance were rather independent of the setting, that is, the findings were very similar for both subject samples (PPMI, clinical), all reconstruction algorithms (OSEM with and without resolution recovery, filtered backprojection), and both ROI methods to estimate the SBR (conventional ROI analysis, hottest voxels analysis). Given that the settings considered here are quite different (Figs. 1, 2, 3), the robustness of the results with respect to the setting suggests that these findings hold more generally in the spectrum of settings encountered in clinical routine.

**Fig. 6:**
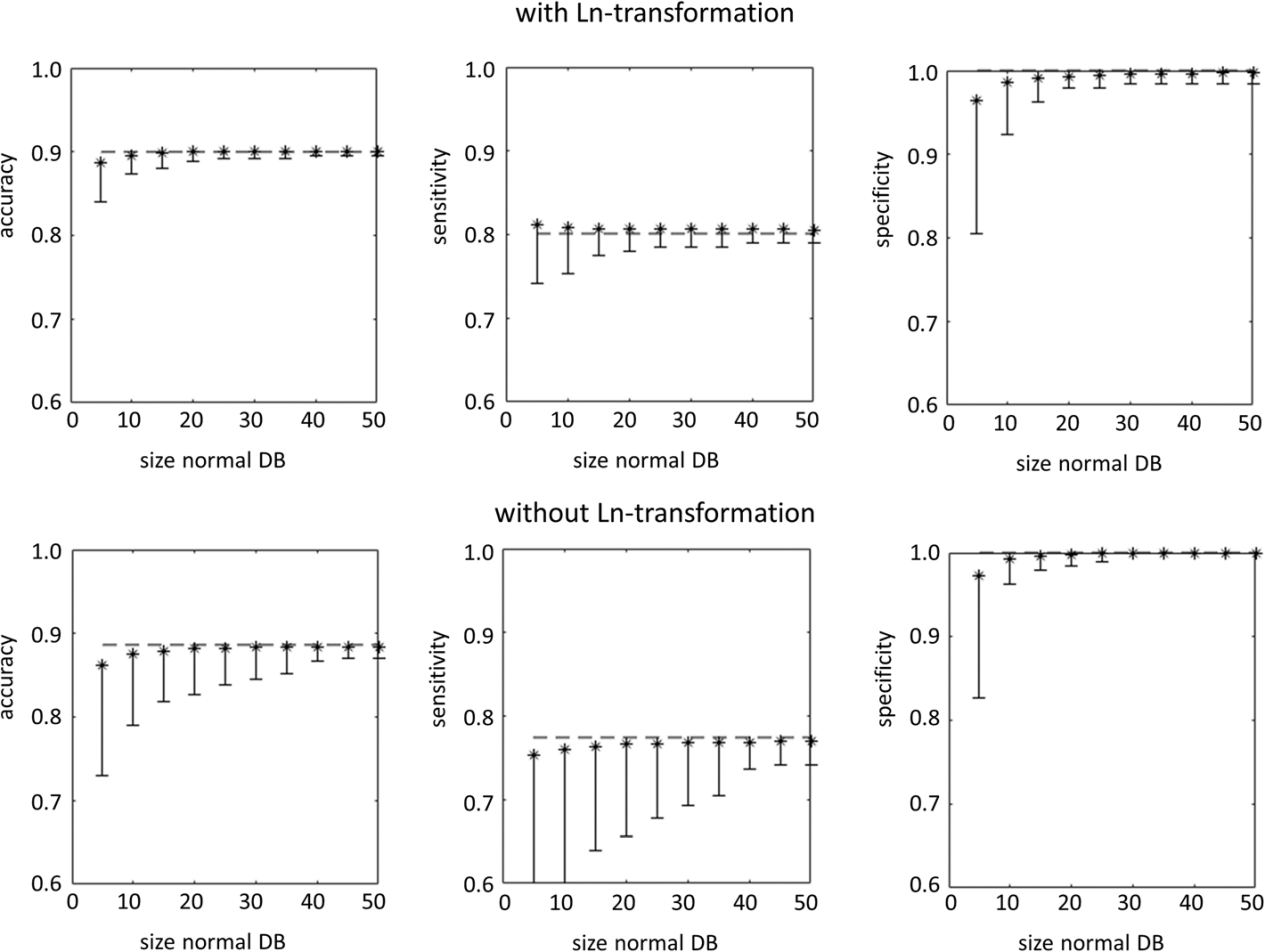
Mean accuracy (left column), sensitivity (middle column), and specificity (right column) of the hottest voxels putamen SBR for identification of the patients with neurodegenerative PS in the clinical sample with iterative OSEM reconstruction and resolution recovery (= setting with largest skewness of the SBR distribution). The top row shows the performance with Ln-transformation, the bottom row without Ln-transformation. The error bars indicate the difference between mean accuracy, sensitivity, or specificity and the 5th percentile over the 10,000 randomly sampled normal databases. The dashed line represents the performance of the z-score of the putamen SBR when all patients with non-neurodegenerative PS (*n* = 186) were used to estimate mean and standard deviation of normal putamen SBR for transforming SBR values into z-scores as benchmark

A secondary finding of this study was the skewness of the distribution of the putaminal SBR in normal DAT SPECT scans that could be reduced by Ln-transformation of the SBR values (Fig. 3). Ln-transformation prior to transformation to z-scores improved the classification performance of the putamen SBR (Fig. 6). The improvement of overall accuracy was mainly driven by improved sensitivity (Fig. 6), most likely due to avoiding overestimation of the standard deviation of normal SBR from skewed distributions. The effect of the Ln-transformation was larger for the 5th percentile than for the average performance over the 10,000 random realizations of the normal database, suggesting that the main benefit from Ln-transformation was stabilization of classification performance by reducing the impact of potential outliers in the normal database. In line with this, the decline of the “maximum” performance loss with increasing size of the normal database was faster with Ln-transformation than without. As a consequence, without Ln-transformation, a larger normal database might be required to reliably achieve the same level of performance of the putamen SBR z-score than with Ln-transformation. In general, estimates of mean and standard deviation of normal putamen SBR derived from the normal database are the more sensitive to outliers the smaller the database. Thus, careful control of the DAT SPECT images to be included in the normal database is particularly important in case of a small database.

**Fig. 7:**
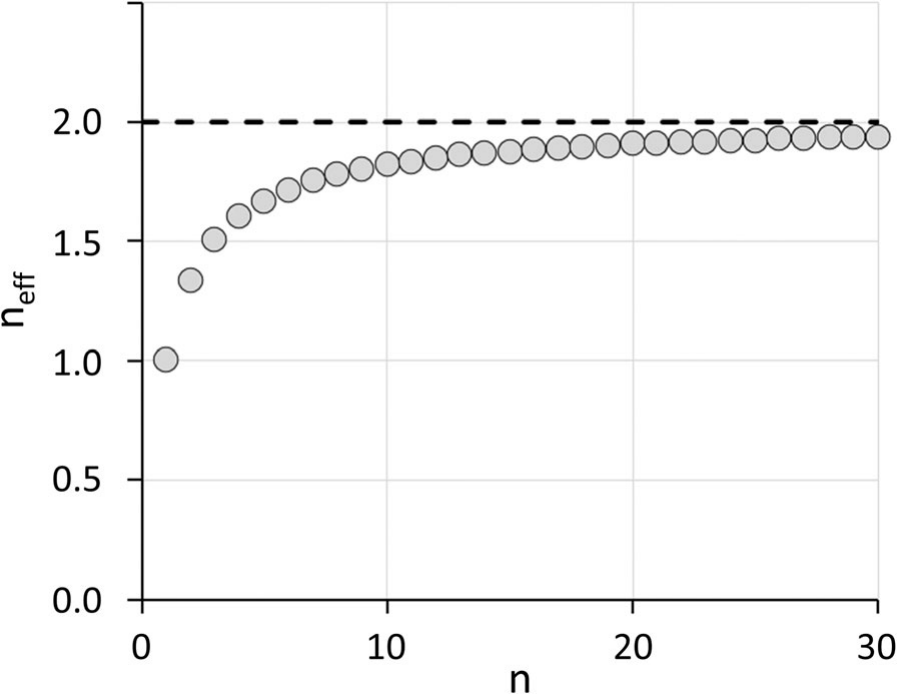
Effective sample size *n*_eff_ versus size *n* of the normal database. The dashed line indicates the limit of *n*_eff_ approached at very large *n*

This study focused on DAT SPECT with FP-CIT. In order to discuss potential generalizability of the findings, one might hypothesize that univariable binary classification of FP-CIT SPECT, that is, differentiation between neurodegenerative and non-neurodegenerative PS using the putaminal SBR, can be considered a two-sample t-test with the single subject to be classified comprising one group and the normal database comprising the other group. Furthermore, the statistical power of testing a given feature for a mean difference between two groups of different size (*n*_1_, *n*_2_) is approximately equal to the power of comparing it between two groups of equal size *n*_eff_ with *n*_eff_ = 2 * *n*_1_ * *n*_2_ / (*n*_1_ + *n*_2_) [35]. Assuming this equation to be approximately valid also for the extreme case of single subject comparison against a normal database [36], that is, *n*_1_ = 1 and *n*_2_ = *n* = size of the normal database, it is *n*_eff_ = 2 * *n* / (*n* + 1). The plot of this relation (Fig. 7) shows that *n*_eff_ effectively starts to reach its plateau at about *n* = 10 to *n* = 15. We hypothesize, therefore, that adequate mean accuracy of univariable binary classification in general requires a normal database of at least 10 to 15 subjects. The present finding of less than 1% mean loss of accuracy of the putamen SBR z-score when the normal database included at least 15 subjects is in line with this. Yet, the variability of the accuracy between different realizations of the normal database can still vary considerably at this normal database size (comp. Fig. 5b). The number of additional subjects in the normal database required to achieve adequate stability of the classification performance between different realizations of the normal database depends on the between-subjects variability of the feature used for classification as well as on the mean difference of the feature between disease-positive patients and the normal database (effect size). The number of additional normal subjects required to achieve stable performance between different realizations of the normal database, therefore, is expected to depend on the application. In case of putamen SBR-based classification of FP-CIT SPECT, the number of additional normal subjects required was 10 to 15 (resulting in a total of 25 to 30 subjects in the normal database).

The following limitations of this study should be noted. First, the comparison of filtered backprojection versus iterative OSEM reconstruction was restricted to the clinical sample. The PPMI provides only FP-CIT SPECT images reconstructed with OSEM for download. Raw FP-CIT SPECT projection data for retrospective reconstruction to test other reconstruction algorithms are not available. Furthermore, the OSEM parameters differed between the PPMI sample and the clinical sample. In particular, OSEM reconstruction was performed with resolution recovery in the clinical sample and without in the PPMI sample. The rationale for this was to increase the heterogeneity among the tested settings. Second, the whole sample was used as test set in all cases. The rationale for this was to use the same test set for all sizes of the normal database in order to avoid bias by varying sizes of the test set. As a consequence, the healthy controls (PPMI sample) or the patients with non-neurodegenerative PS (clinical sample) randomly selected for the normal database were also included in the test set. This might have resulted in overly optimistic performance estimates. However, the effect is expected to be small, because the subjects in the normal database represented only a small fraction (< 13.5%) of the test set in all cases. Third, the normal database of the clinical sample was generated retrospectively from patients who had received FP-CIT SPECT for the etiological diagnosis of a clinically uncertain PS in routine patient care. The clinical diagnosis of a non-neurodegenerative etiology (not associated with nigrostriatal degeneration) as standard of truth was based on the written report of a movement disorder specialist in the patient’s file after FP-CIT SPECT. The movement disorder specialist was not blinded for the FP-CIT SPECT findings. This might have caused some bias in favor of FP-CIT SPECT resulting in overly optimistic performance estimates of the putamen SBR in the clinical sample. The potential bias is not expected to affect the evaluation of the impact of the size of the normal database on the performance of the putamen SBR. Fourth, this study used conversion to z-scores and a fixed, predefined cutoff on the z-score for SBR-based classification of FP-CIT SPECT. Other methods to define a cutoff such as receiver operating characteristic (ROC) analysis require a database of patients with nigrostriatal degeneration in addition to a normal database. The impact of the sizes of the two databases (without and with nigrostriatal degeneration) on SBR classification performance using cutoffs derived from ROC analysis might be addressed in future studies. Fifth, neither age nor gender were taken into account in this study, although there is strong evidence for age related decline of striatal DAT availability [37] and moderate evidence for higher striatal DAT availability in females compared to males [38–40]. However, so far no studies have been published that clearly demonstrate that age- and/or gender-correction of the putaminal FP-CIT SBR improves its diagnostic performance [41]. Finally, normal databases of FP-CIT SPECT from healthy control subjects were used for the PPMI settings, whereas normal databases composed of visually normal FP-CIT SPECT from patients with non-neurodegenerative PS were used for the clinical settings. Nevertheless, the present study did not allow testing the impact of the type of the normal database (healthy control subjects versus patients with Parkinsonism but visually normal FP-CIT SPECT) on the performance of semi-quantitative analysis in FP-CIT SPECT. This would require two normal databases for the same setting, one comprised of healthy controls, the other comprised of patients with non-neurodegenerative PS.

## Conclusion

In conclusion, the results of this study suggest that 25 to 30 is the minimum size of the normal database to reliably achieve good performance of semi-quantitative analysis in DAT SPECT, independent of the algorithm used for image reconstruction and the ROI method used to estimate the putaminal SBR. Increasing the size of the normal database beyond 40 provides only very small further improvement.

## Data Availability

The datasets supporting the conclusions of this article can be made available on request.

## Appendix: Other conventional semi-quantitative parameters did not provide additional information beyond putaminal DAT availability

The following other conventional semi-quantitative parameters were tested for identification of patients with neurodegenerative PS in the clinical sample (with filtered backprojection) using receiver operating characteristic (ROC) analysis: minimum of left and right caudate SBR, minimum of left and right putamen-to-caudate SBR ratio, left-right asymmetry of putamen SBR (= 200 * abs (left − right) / (left + right)), and left-right asymmetry of caudate SBR. The clinical sample was used for this purpose in order to avoid the ceiling effect in the PPMI sample (due to very good performance of the putaminal SBR alone in the PPMI sample).

The area under the ROC curve was significantly smaller for each of the other conventional semi-quantitative parameters than for the (minimum of left and right) putamen SBR (0.953 ± 0.011): 0.932 ± 0.012 (DeLong *p* = 0.017), 0.886 ± 0.018 (*p* < 0.001), 0.824 ± 0.023 (*p* < 0.001), and 0.730 ± 0.027 (*p* < 0.001) for caudate SBR, putamen-to-caudate SBR ratio, left-right asymmetry of putamen SBR, and left-right asymmetry of caudate SBR, respectively.

Discriminant analysis was performed in order to test whether one or more of the other parameters might provide additional diagnostic information beyond the putamen SBR.

Stepwise discriminant analysis (Wilks’ method, entry *p* = 0.05, removal *p* = 0.10) did not include any of the other parameters (*p* = 0.699, 0.596, 0.226, and 0.341 for caudate SBR, putamen-to-caudate SBR ratio, left-right asymmetry of putamen SBR, and left-right asymmetry of caudate SBR, respectively). When the analysis was forced to include all other parameters, the resulting discriminant function was as follows:

Discriminant function = − 3.741 + 3.200 * putamen SBR + 0.105 * caudate SBR + 0.622 * putamen-to-caudate ratio − 0.590 * putamen asymmetry / 100 − 0.309 * caudate asymmetry / 100

ROC analysis of the discriminant function revealed exactly the same area under the ROC curve as for the putamen SBR alone. Thus, there was no evidence that the other conventional semi-quantitative parameters provide additional information beyond the putaminal SBR that might improve the performance of semi-quantitative analysis of FP-CIT SPECT in the clinical sample.

This does not rule out that the other conventional semi-quantitative parameters might provide additional information beyond the putaminal SBR in other patient samples, for example, in samples with more borderline cases or a larger fraction of atypical neurodegenerative PS (multiple system atrophy, progressive supranuclear palsy, corticobasal degeneration), or in the differentiation between dementia with Lewy bodies and Alzheimer’s disease [42].

## Abbreviations

AAL: Automatic Anatomic Labeling
DAT: Dopamine transporter
FBP: Filtered backprojection
FP-CIT: N-ω-fluoropropyl-2β-carbomethoxy-3β-(4-I-123-iodophenyl)nortropane
HC: Healthy controls
HV: Hottest voxels
Ln: Log natural
MNI: Montreal Neurological Institute
OSEM: Ordered subsets expectation maximization
PD: Parkinson’s disease
PPMI: Parkinson’s Progression Marker Initiative
PS: Parkinsonian syndrome
ROI: Region of interest
RR: Resolution recovery
SBR: Specific binding ratio
SPECT: Single-photon emission computed tomography
SPM12: Statistical Parametric Mapping software package, version 12
